# Mendelian randomization in (epi)genetic epidemiology: an effective tool to be handled with care

**DOI:** 10.1186/s13059-016-1018-9

**Published:** 2016-07-14

**Authors:** Antti Latvala, Miina Ollikainen

**Affiliations:** Finnish Twin Cohort Study, Department of Public Health, University of Helsinki, 00290 Helsinki, Finland; Institute for Molecular Medicine Finland, FIMM, University of Helsinki, 00290 Helsinki, Finland

## Abstract

A study examining blood lipid traits takes epigenomics approaches to the next level by using carefully performed Mendelian randomization to assess causality rather than simply reporting associations.

See related research article: http://genomebiology.biomedcentral.com/articles/10.1186/s13059-016-1000-6

Epidemiology is concerned with identifying modifiable etiological factors for health outcomes in the population, but it faces the challenge of distinguishing causal influences from mere statistical associations. Causality between a risk factor or exposure *X* and a trait or disease outcome *Y* can be established by conducting an experiment; however, in many cases ethical and practical limitations rule this out. Researchers must then rely on observational studies to establish associations between exposures and outcomes, but these associations might not reflect true causal effects owing to the inability to exclude possible alternative mechanisms.

When genetic variations are studied as exposures, reverse causality is ruled out because the DNA sequence remains unchanged throughout life (although the genetic markers might not be the actual causal variants but only in linkage disequilibrium (LD) with them [1, 2]). When epigenetic states are investigated as mediating mechanisms between exposures and health outcomes, the challenges of causal inference equal those faced in traditional epidemiological studies. Epigenetic states are unstable and tissue specific; thus epigenetic differences between cases and controls could be either causes or consequences of the disease, or arise owing to confounding factors. In a new study, Dekkers and colleagues [3] show how a Mendelian randomization approach can be used to address these challenges.

## Mendelian randomization as a tool for causal inference

Even if real experiments are not possible, various quasi-experimental designs could still be available to aid in testing causal hypotheses. One such design is Mendelian randomization (MR), which has become well known in observational epidemiology [4, 5]. MR applications for epigenetic epidemiology have been developed [6] and have recently been applied by Dekkers and colleagues to study causality between blood lipids and DNA methylation in circulating cells [3].

**Fig. 1:**
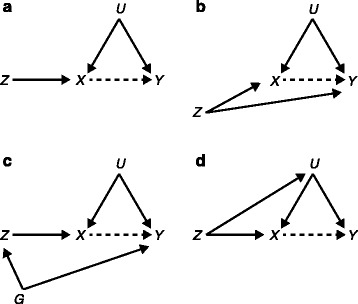
**a** Illustration of Mendelian randomization to study causality between exposure and outcome in the presence of confounders. **b** Illustration of a situation when the assumptions of Mendelian randomization are violated because the genetic instrument has pleiotropic effects, influencing both the exposure and the outcome. **c** The assumptions are violated because the genetic instrument is in linkage disequilibrium with another variant that is associated with the outcome. **d** The genetic instrument is associated with confounding factors, violating the assumptions of Mendelian randomization. *X*, modifiable exposure of interest; *Y*, outcome of interest; *Z*, genetic variant (a single allele or a linear combination of several alleles) used as an instrumental variable; *U*, (measured or unmeasured) confounding factors; *G*, genetic variant that is in linkage disequilibrium with *Z*. The *broken arrow* denotes an assumed, but unknown, causal effect

MR is a form of instrumental variable analysis and is based on the concept that, if *X* affects *Y*, factors affecting *X* must also have an effect on *Y*. In MR, a genetic variant *Z* that is known to associate with *X* is utilized as an ‘instrumental variable’ to investigate the causal nature of the association between *X* and *Y* (Fig. 1a). If the underlying assumptions (see below) are met, an association between *Z* and *Y* can be taken as evidence for causality between *X* and *Y* [4]. The random segregation and assortment of alleles from parent to offspring during gamete formation ensures that associations between genetic variation and outcomes of interest are usually not susceptible to confounding. Because an individual’s genotype precedes the outcome, reverse causality is also not an issue. The segregation of alleles during meiosis can thus be seen as analogous to the randomization process in randomized controlled trails (RCTs) [4].

While the scope of applications is limited by its assumptions, MR has been applied to various exposures, such as cholesterol levels, alcohol intake, body mass index, and C-reactive protein, with varying findings regarding their effects on diverse outcomes, such as cardiovascular disease, Alzheimer’s disease, exercise levels, and depression [5]. Epigenetic applications are still relatively rare but include studies of maternal hyperglycemia affecting epigenetic regulation of the leptin gene in newborns [7] and methylation near the gene encoding interleukin-4 influencing serum immunoglobulin E levels [8].

## Assumptions and pitfalls of Mendelian randomization

Causal inferences drawn from MR studies are only valid if the rather strict assumptions of the method are met [4]. First, the genetic instrument *Z* must be associated with the exposure *X*. This must be a reliably established association, not merely based on the study sample at hand. Second, the genetic instrument *Z* must not be associated with the confounding factors *U*. Third, the genetic instrument *Z* should only be related to the outcome *Y* through its association with the exposure *X*. Figure 1a shows a directed acyclic graph of the situation when these assumptions hold.

The first assumption is the least problematic. Indeed, large-scale genome-wide association studies (GWASs) have produced large numbers of rather reliable gene–trait associations [1, 2]. Assuming that a genetic instrument (either a single allele or a linear combination of several alleles) for *X* is available, the remaining assumptions become crucial. As has been pointed out, it is never possible to prove definitively that these assumptions hold. Instead, their validity must be weighed based on biological knowledge [4, 5].

If the genetic instrument is directly (or via some intervening variable other than *X*) associated with the outcome of interest, MR will produce incorrect or at least biased results because an observed association between *Z* and *Y* would not only tell about the association between *X* and *Y*. This would happen if the genetic variant had pleiotropic effects, influencing not only *X* but also *Y*, as in Fig. 1b. Notably, recent genome-wide analyses point towards significant pleiotropic effects for different health outcomes [1, 2]. The situation is more challenging when polygenic scores (PSs) combining several alleles are used as instruments. Crucially, in this situation, the assumptions of MR should hold for all variants included in the allele score [9]. Thus, for unbiased MR results, none of the included alleles should have a pleiotropic effect on the outcome *Y*. This can be problematic for studies using PSs as instruments—which is increasingly common as the GWAS approach points towards highly polygenic effects for complex traits—if pleiotropy turns out to be more widespread than has been previously thought. Recently, multivariable MR has been developed to address pleiotropy [5].

Besides pleiotropic effects, LD between the genetic instrument *Z* and some other allele(s) that influence *Y* would violate the assumptions of MR (Fig. 1c). While the LD structure is generally known, using a PS as an instrument complicates the situation here also. Finally, the assumptions would be violated also by a pleiotropic effect on (or LD with a pleiotropic variant on) the confounding factors *U* (Fig. 1d). For example, socioeconomic status is an important confounder for many health outcomes. A recent GWAS identified 74 loci associated with educational attainment, many of them in regions regulating gene expression in the brain [10]. Thus, if researchers were to include some of these loci in a PS genetic instrument for some *X* for which educational level is a confounder, bias might be introduced through the association of the genetic instrument and educational attainment.

Furthermore, even if the assumptions of MR hold, there are additional limitations. One potential concern is that genetic variants (including PSs) are often only weakly associated with exposures of interest, making them weak instruments [4]. This is reflected in imprecise estimates, necessitating large samples for adequately powered studies. However, in addition to this statistical limitation, there is a potential biological concern: do we expect the (typically) small change in *X*, induced by *Z*, to have a biologically meaningful effect on *Y*?

Despite the underlying assumptions and potential limitations, MR is a promising tool for causal inference in (epi)genetic epidemiology, as is illustrated by the study of Dekkers and colleagues [3]. These authors interrogated the causal relationship between blood lipids and genome-wide DNA methylation, and show that differential methylation is the consequence, rather than the cause, of inter-individual variation of blood lipids. To do this, they used a clever strategy that involved performing MR in a stepwise manner.

## MR as a tool to infer causality between blood lipid levels and DNA methylation

The first step taken by Dekkers and colleagues was to establish reliable instrumental variables. Recent GWASs for blood lipid levels had shown robust associations between 40, 57, and 69 single nucleotide polymorphisms (SNPs) with levels of triglycerides, low-density lipoprotein cholesterol (LDL-C), and high-density lipoprotein cholesterol (HDL-C), respectively, providing reliable instrumental variables (i.e., unbiased predictors of lipid levels) for a MR to test for causality. Instead of using single SNP genotypes, Dekkers et al. generated PSs to increase the effect size. Still, however, the effect sizes were as small as around 5 % for each lipid, leading to rather low power in the MR analysis.

The next step was to establish lipid–methylation associations. The authors performed three epigenome-wide association studies (EWASs) to identify CpG sites whose methylation associates with triglycerides, LDL-C, and HDL-C. This was performed in a population of 3296 individuals, which is a considerably large population in the current EWAS era. With the EWAS, they showed robust associations with small effect sizes, common for any non-cancer EWAS, between CpG methylation and lipid levels in six Dutch cohorts. They identified 21 CpGs whose methylation associated with triglyceride levels, three with LDL-C, and four with HDL-C, and replicated some of the previous EWASs on blood lipids and associated traits.

The third step was to remove direct associations. Genotypes have a large effect on DNA methylation variation both globally and locally. SNPs that affect local DNA methylation are called methylation quantitative trait loci (meQTL). As the assumption in the MR is that the effect of a SNP upon CpG methylation is mediated through lipid levels, all identified direct effects between SNPs and DNA methylation (i.e., meQTL) at the lipid-associated CpG sites were discarded.

An additional step was to test for reverse causation. This was addressed by using a SNP in *cis* for each MR-identified CpG as a proxy for DNA methylation. No evidence for methylation affecting the lipid levels was observed—thus, reverse causation was unlikely.

Testing for pleiotropy was also an important step of this study. As pleiotropy is a common confounder in MR analyses, especially when using PSs as instrumental variables, the possibility of each SNP affecting the levels of multiple lipids was addressed. Here, multivariable MR and Egger regression were successfully applied to identify any pleiotropic effects.

Finally, this carefully designed stepwise MR analysis resulted in a small number of CpG sites whose methylation was affected by the circulating lipid levels, and that associated with the expression of the respective gene that the CpG site was located in. All these genes have a role in lipid metabolism.

## Concluding remarks

MR approaches provide useful tools to infer causality in (epi)genetic epidemiology. However, MR studies are only valid if the rather strict assumptions of the method are met. In addition, it is never possible to prove definitively that these assumptions hold. Instead, their validity must be weighed based on prior biological knowledge. Thus, Mendelian randomization is an effective tool in (epi)genetic epidemiology, but the method needs to be handled with care. Ideally, causality in epigenetic analyses should be supported by multiple independent approaches with different assumptions, including animal models, longitudinal studies of exposure-discordant monozygotic twins, and MR.

## Abbreviations

EWAS, epigenome-wide association study; GWAS, genome-wide association study; HDL-C, high-density lipoprotein cholesterol; LD, linkage disequilibrium; LDL-C, low-density lipoprotein cholesterol; meQTL, methylation quantitative trait loci; MR, Mendelian randomization; PS, polygenic score; RCT, randomized controlled trial; SNP, single nucleotide polymorphism
